# High-responsivity hybrid α-Ag_2_S/Si photodetector prepared by pulsed laser ablation in liquid

**DOI:** 10.3762/bjnano.11.142

**Published:** 2020-10-21

**Authors:** Raid A Ismail, Hanan A Rawdhan, Duha S Ahmed

**Affiliations:** 1 Department of Applied Science, University of Technology, Baghdad, Iraq

**Keywords:** cetyltrimethylammonium bromide (CTAB), laser ablation, monodisperse, photodetector, silver(I) sulfide (Ag_2_S), thiourea

## Abstract

We report the synthesis of α-Ag_2_S nanoparticles (NPs) by one-step laser ablation of a silver target in aqueous solution of thiourea (Tu, CH_4_N_2_S) mixed with cationic cetyltrimethylammonium bromide (CTAB) as surfactant. The effect of the CTAB surfactant on the structural, morphological, optical, and elemental composition of Ag_2_S NPs was evaluated using X-ray diffraction (XRD), transmission electron microscopy (TEM), scanning electron microscopy (SEM), energy-dispersive X-ray spectroscopy (EDX), and UV–vis spectroscopy. The optical absorption decreased and the optical energy gap of α-Ag_2_S increased from 1.5 to 2 eV after the CTAB surfactant was added to the Tu solution. XRD studies revealed that the synthesized Ag_2_S NPs were polycrystalline with a monoclinic structure and that crystallinity of the nanoparticles was improved after adding CTAB. Raman studies revealed the presence of peaks related to Ag–S bonds (A_g_ modes) and the longitudinal optical phonon 2LO mode. Scanning electron microscopy investigations confirmed the production of monodisperse Ag_2_S NPs when using the CTAB surfactant. The optoelectronic properties of α-Ag_2_S/p-Si photodetector, such as current–voltage characteristics and responsivity in the dark and under illumination, were also improved after using the CTAB surfactant. The responsivity of the photodetector increases from 0.64 to 1.85 A/W at 510 nm after adding CTAB. The energy band diagram of the α-Ag_2_S/p-Si photodetector under illumination was constructed. The fabricated photodetectors exhibited reasonable stability after three weeks of storage under ambient conditions with a responsivity of 70% of the initial value.

## Introduction

Nanomaterials have attracted considerable attention due to their superior chemical and physical properties. The size-dependent properties of nanomaterials have enabled them to be used in many promising applications, for example, catalysis and electronic and optoelectronic devices [1–3]. In this regard, controlling particle sizes and attaining them within a narrow size distribution are important. Silver sulfide is an important semiconducting material with a narrow direct optical energy gap, which ranges from 0.96 to 1.1 eV at room temperature. Ag_2_S has good chemical stability, low toxicity, and high optical absorption [4]. According to the growth temperature, Ag_2_S has three phases: monoclinic α-Ag_2_S (acanthite), β-Ag_2_S (argentite), and the stable γ-Ag_2_S [5–6]. Silver sulfide nanoparticles (NPs) are extensively used in many applications, such as photoconductors, solar cells, infrared (IR) photodetectors, biosensors, photocatalysts, and probes [7–9]. A number of techniques have been used to synthesize nanostructured Ag_2_S, including facile hydrothermal methods, chemical bath deposition, laser ablation in liquid reverse microemulsion, electrospinning, sol–gel, electrochemical method, template method, sonochemical method, and hydrochemical bath deposition [10–13]. The size of Ag_2_S NPs depends on the preparation conditions [14]. Ag_2_S NPs show a strong tendency to agglomerate and aggregate and consequently form large particles. In this context, extensive studies have been conducted to obtain monodisperse and single-morphology Ag_2_S NPs. Dong et al. prepared faceted and cubic Ag_2_S nanocrystals using a cost-effective cetyltrimethylammonium bromide (CTAB) surfactant-assisted hydrothermal method [15]. Zhang et al. synthesized monodisperse Ag_2_S NPs using thermolysis of harmless silver xanthates as a single-source molecular precursor and controlled the particle size by changing the alkyl chain length in the precursors [16]. Recently, Kang et al. synthesized monodisperse Ag_2_S NPs by using a sonochemical method and fabricated photodetector devices by integrating Ag_2_S NPs on a graphene sheet [17]. Tretyakov et al. [18] reported the characterization of a Ag_2_SQD (quantum dots)/Si heterojunction photodetector used for short-wave infrared radiation fabricated by a chemical method. They show that the Ag_2_S quantum dots (QDs) planted on the surface of Si create impurity states in the Si bandgap. In pulsed laser ablation, the interaction between laser and material particles leads to severe particle aggregation and broad particle size distributions via melting/fragmentation [19]. In the present work, we demonstrate a novel technique to prepare monodisperse Ag_2_S NPs using CTAB surfactant-assisted pulsed laser ablation of Ag_2_S NPs in a thiourea (Tu) solution. Moreover, a high-performance hybrid Ag_2_S/Si photodetector was fabricated.

## Experimental

**Figure 1:**
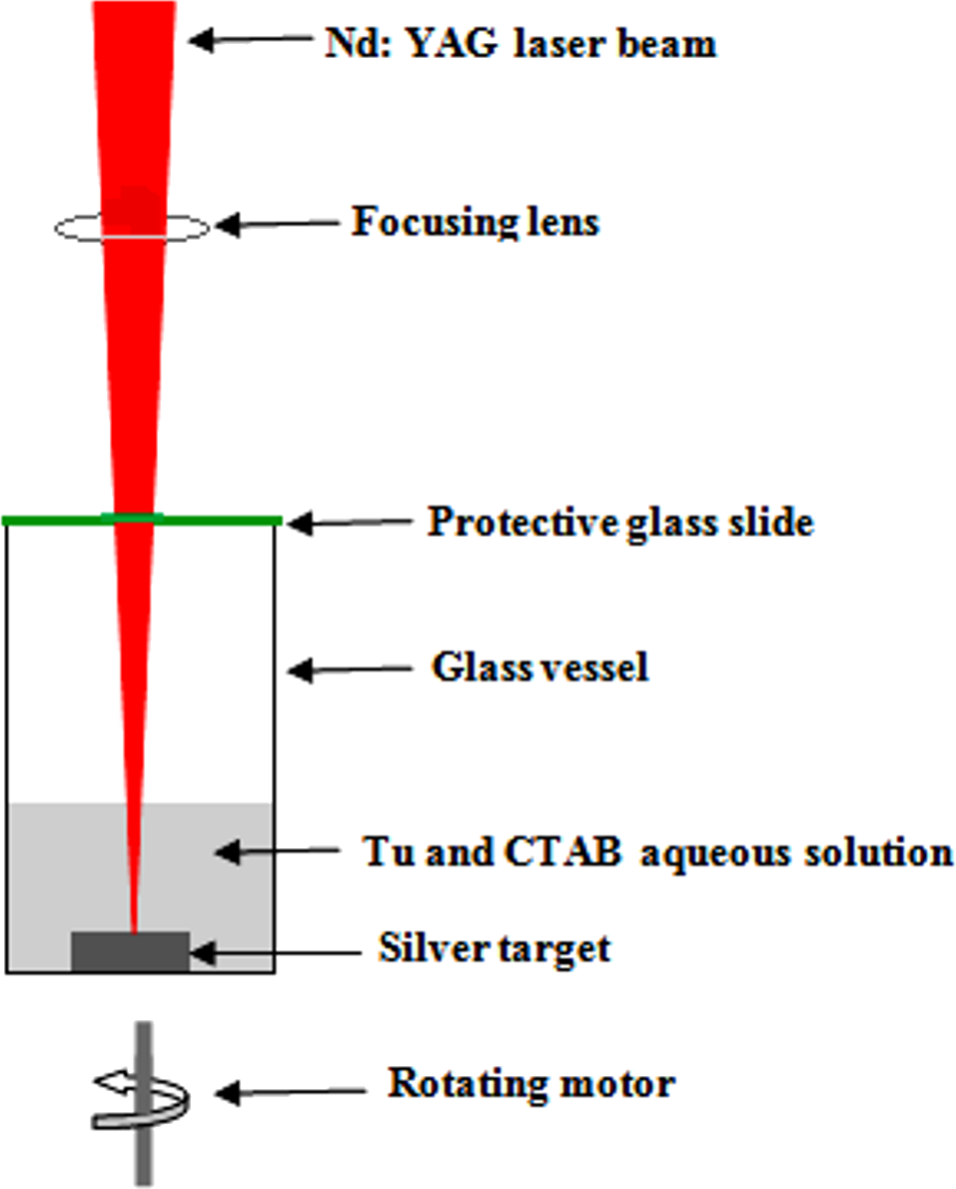
Schematic diagram of PLAL system used for preparation of Ag_2_S NPs.

Colloidal Ag_2_S NPs were prepared by laser ablation of a high-purity silver target in an aqueous solution of thiourea (Tu, CH_4_N_2_S) mixed with an aqueous solution of the surfactant cetyltrimethylammonium bromide (CTAB, C_19_H_42_BrN). To prepare the ablation liquid, 0.39 g of Tu was dissolved in 10 mL of double-distilled water (DDW) and then mixed with 0.18 g of CTAB dissolved in 10 mL of DDW. The laser used here was a Q-switched Nd:YAG laser operating at λ = 1064 nm, 7 ns pulse width, and 2 Hz pulse repetition frequency. A high-purity silver (99.9%) pellet with a thickness of 10 mm and a diameter of 20 mm was positioned at the bottom of a glass vessel filled with Tu solution mixed with the CTAB solution. The height of the solution was 2 mm above the Ag target. The laser beam was focused on the Ag pellet by using a converging lens of 10 cm focal length. The laser fluence used for ablation was 3.9 J·cm^−2^·per pulse taking into account the transmittance of the ablation liquid at 1064 nm. The ablation time for each sample was set to 20 min. Figure 1 shows a schematic of the pulsed laser ablation system used in this work. A rotating motor was used to help prevent the aggregation and agglomeration of particles during the ablation process. The vessel was covered with a thin glass slide to prevent the vapor from reaching the laser focusing lens. The optical absorbance of the colloidal Ag_2_S NPs was measured by using a UV–vis double-beam spectrophotometer (Lambda 750, Perkin Elmer). The thiourea solution was used as reference in one cuvette and the second cuvette was filled with thiourea solution and Ag_2_S nanoparticles. An X-ray diffractometer (XRD-6000, Shimadzu) was used to investigate the structural properties of Ag_2_S NPs deposited on the glass substrate. A Fourier-transform IR (FTIR) spectrophotometer (8400S, Shimadzu) was employed to estimate the chemical composition of the Ag_2_S NPs. The morphology and size of the nanocrystals were examined via transmission electron microscopy (TEM; EM208, Philips). Raman spectroscopy was performed on colloidal Ag_2_S and thiourea solution using a Raman spectrometer (Bruker Optics, Germany). An Ag_2_S/Si photodetector was prepared by depositing a Ag_2_S layer on the front side of a silicon substrate through a mask by drop-casting. A single-crystal p-type silicon (111) substrate with an electrical resistivity of 3–5 Ω·cm and a thickness of 300 μm was used.

**Figure 2:**
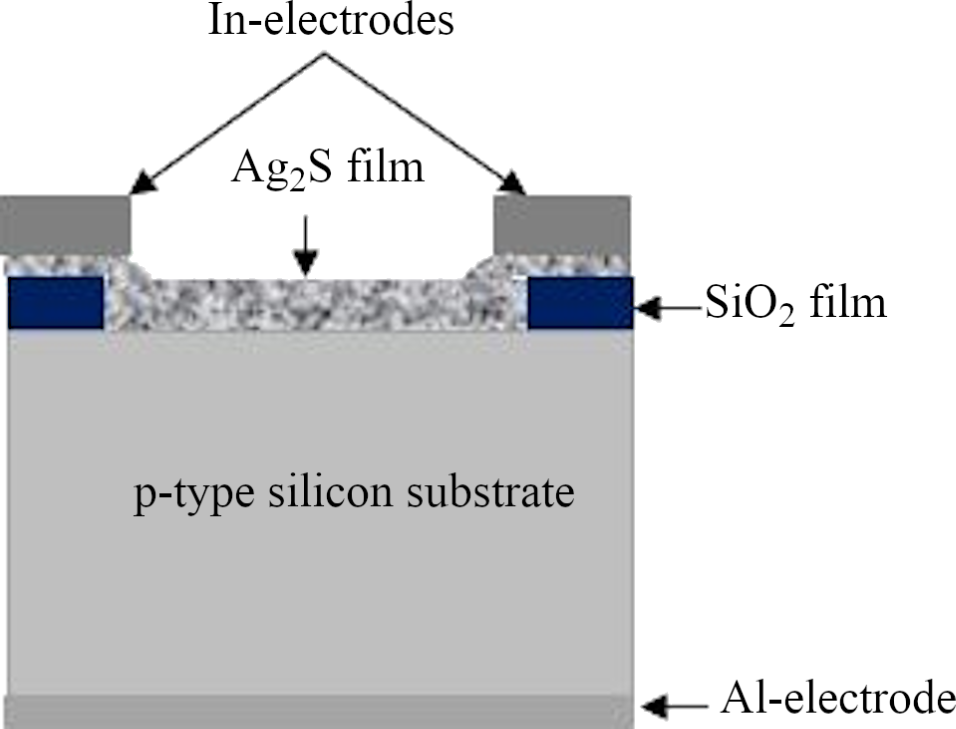
Cross-sectional view of the n-Ag_2_S/p-Si heterojunction photodetector.

As shown in Figure 2, a SiO_2_ thin film was grown on the silicon substrate before Ag_2_S deposition through rapid thermal oxidation (RTO) at a temperature of 950 °C for 25 s, and then HF etchant was used to open a Si window on SiO_2_. The experimental details regarding the RTO process are presented elsewhere [20]. To investigate the optoelectronic properties of the Ag_2_S/Si photodetector, ohmic contacts were made by thermally evaporating In and Al films on the nanostructured Ag_2_S film and the back side of the silicon substrate, respectively, as shown in Figure 2.

The sensitive area of the planar photodetector was 1.5 cm^2^. The current–voltage (*I*–*V*) characteristics of Ag_2_S/Si under dark and illuminated conditions were investigated at room temperature using a digital power supply, an electrometer, and a tungsten lamp. The spectral responsivity of the photodetector was measured using a calibrated monochromator in the spectral region of 350–900 nm. The power calibration was performed using accurate silicon power meter (Sanwa).

## Results and Discussion

The formation of Ag_2_S NPs can be described as follows: When the high intensity of the laser beam irradiates the silver target, the absorbed energy results in lattice vibration and silver material is expelled from the target surface in the form of a plasma plum. Thus, silver ions Ag^+^ and sulfur ions S^2−^ are produced from silver target and thiourea solution, respectively. They form Ag_2_S NPs according to the following chemical reaction [21]:

[1]
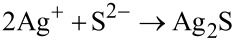


**Figure 3:**
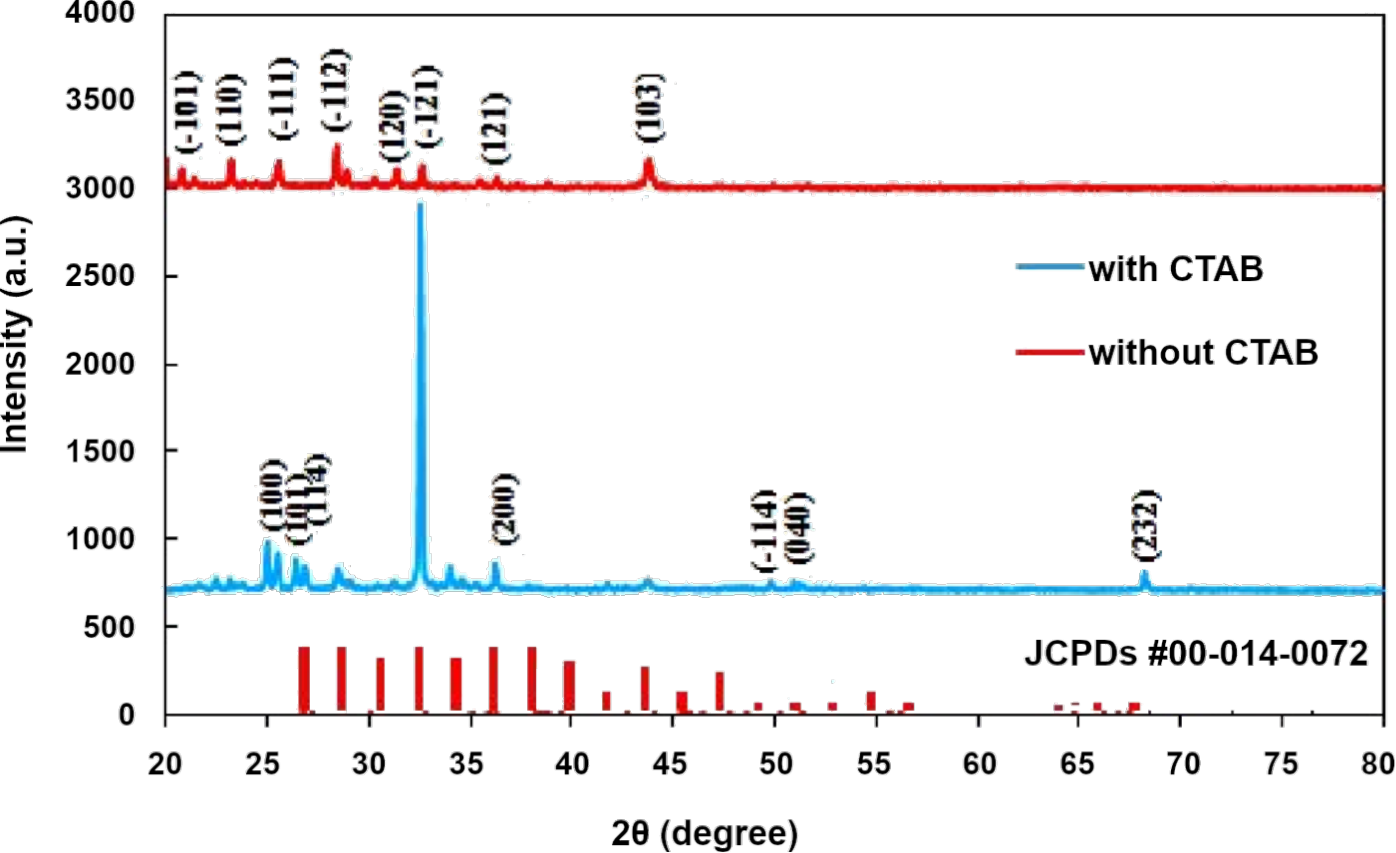
XRD patterns of monoclinic Ag_2_S NPs synthesised with CTAB and without CTAB. The XRD lines are represent the XRD pattern of Ag_2_S powder (JCPDs #00-014-0072).

Figure 3 shows the XRD patterns of Ag_2_S NPs prepared with and without CTAB. The XRD pattern of the Ag_2_S prepared in pure Tu exhibited peaks at 2θ = 24.5°, 25.57°, 26.92°, 32.57°, and 34.57°, which correspond to the (−101), (110), (−111), (−112), (120), (−121), (121), and (103) planes, respectively, of monoclinic Ag_2_S, according to JCPDs Card #00-014-0072 [22]. The XRD pattern of the silver sulfide NPs prepared in Tu and CTAB solution displayed several new XRD peaks at 2θ = 50.4°, 52.6°, and 68.20°, which correspond to the (−114), (040), and (232) planes, respectively, of monoclinic Ag_2_S. No shift in the diffraction peaks was detected after adding the CTAB surfactant. However, a preferred orientation along the (−121) plane was found when the CTAB surfactant was used. The significantly increased intensity of the (−121) plane and other peaks could be attributed to the increase in the degree of crystallinity of Ag_2_S caused by the addition of the CTAB surfactant [23]. The presence of these new peaks was due to the addition of the surfactant, which modified the morphology of the structure. The NPs became nearly spherical, as described in the TEM analysis. These results revealed that the cationic surfactant CTAB substantially influenced the formation of the Ag_2_S NPs. The calculated lattice constants of the Ag_2_S NPs without CTAB were *a* = 4.57 Å, *b* = 6.8 Å, and *c* = 7.89 Å, and those with CTAB were *a* = 4.3 Å, *b* = 6.85 Å, and *c* = 7.8 Å. These values agree well with reported data about monoclinic Ag_2_S. The crystallite sizes of the Ag_2_S prepared in the Tu solution with and without CTAB were calculated using Scherrer equation with the most prominent (−121) plane and found to be 26 and 35 nm, respectively. The particle size of Ag_2_S prepared with CTAB was smaller than of that prepared without CTAB, which is in good agreement with reported data [24].

**Figure 4:**
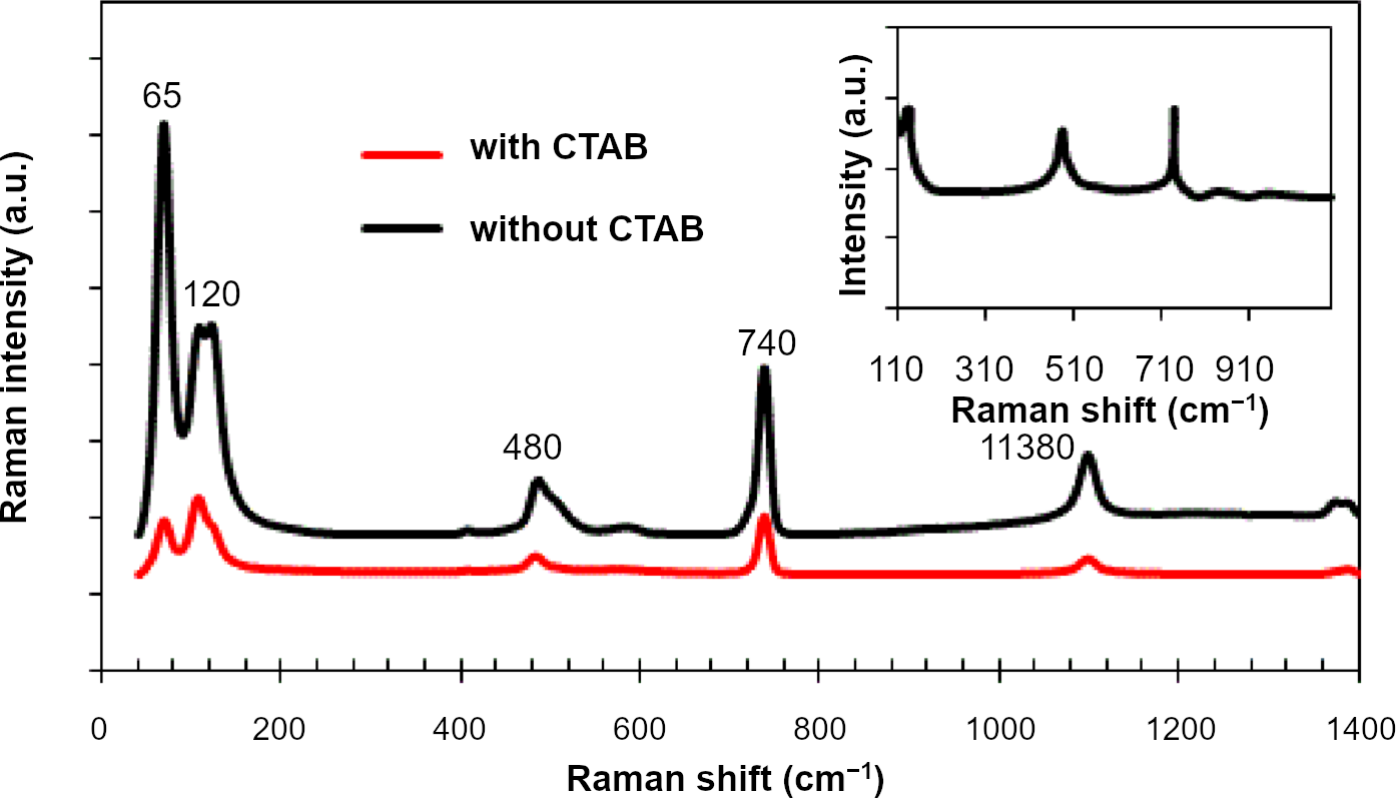
Raman spectra of Ag_2_S NPs prepared without and with CTAB. Inset is the Raman spectrum of thiourea solution.

**Figure 5:**
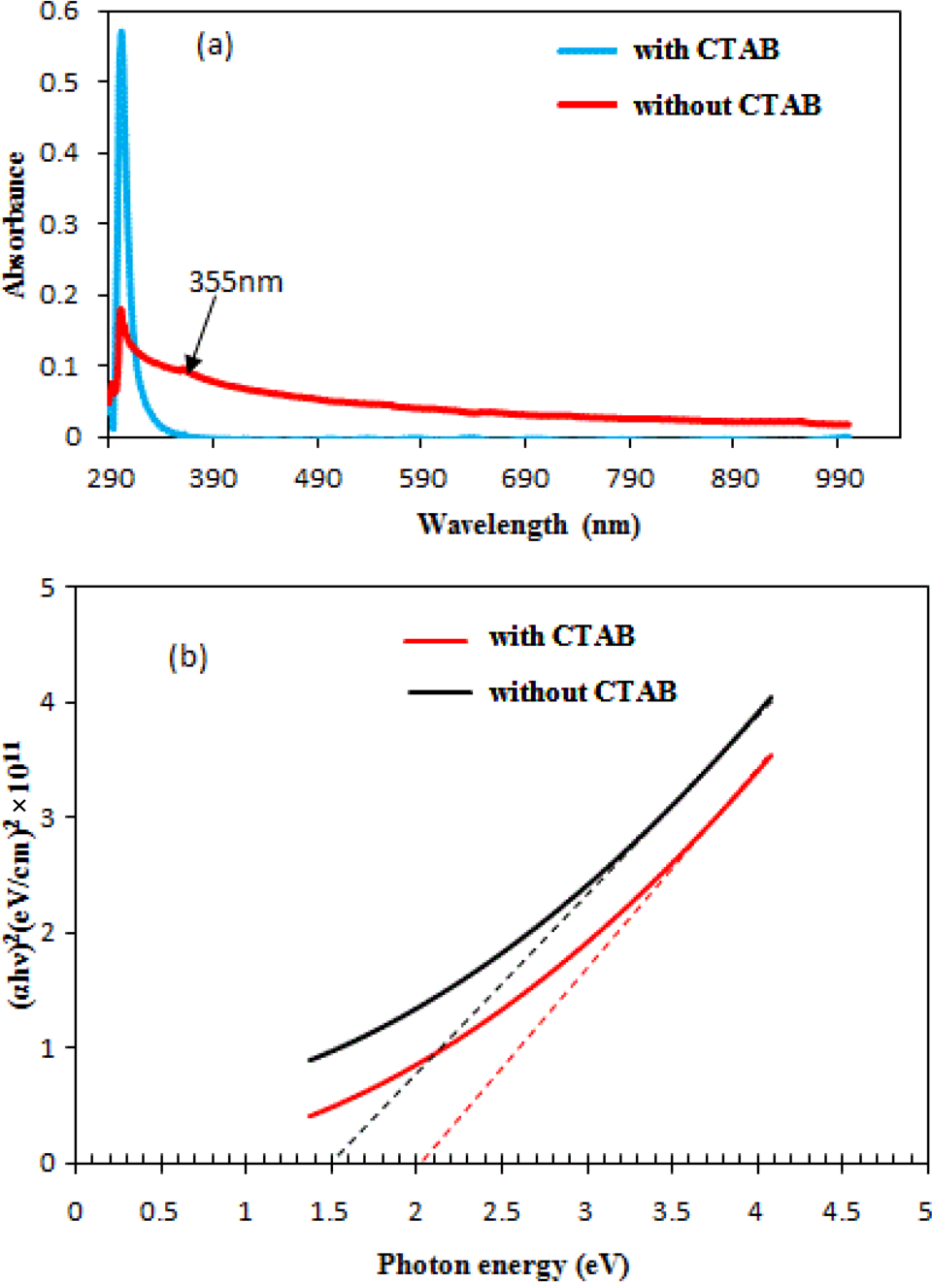
(a) Optical absorption of Ag_2_S prepared with and without CTAB, (b) (α*h*ν)^2^ versus photon energy plot.

Figure 4 shows the Raman spectra of Ag_2_S NPs synthesized in Tu solution with and without CTAB. Four vibration modes were assigned to Ag_2_S. The peaks at 45 and 65 cm^−1^ are related to Ag–S bonds (A_g_ modes) [25]. The third peak at 480 cm^−1^ was indexed to the longitudinal optical phonon 2LO mode in Ag_2_S. The fourth Raman peak, which was at 1380 cm^−1^, was due to the photodecomposition of the Ag_2_S NPs [26–28]. The peak observed at 730 cm^−1^ can be indexed to thiourea as shown in the inset of Figure 4. The Raman spectrum of the particles prepared with CTAB showed an increase in the intensity of peaks (surface-enhanced Raman scattering) due to the reduced agglomeration and aggregation of particles and the small size of the Ag_2_S particles prepared under the effect of the CTAB surfactant [29]. Inset of Figure 4 is the Raman spectrum of thiourea solution, in which three peaks were observed at 125, 484 and 730 cm^−1^. Figure 5a shows the effect of adding CTAB on the UV–vis absorption spectrum of colloidal Ag_2_S NPs. The optical absorption was measured immediately after laser ablation. The addition of the cationic surfactant CTAB to the Tu solution led to a decrease in the optical absorption compared with that prepared in the pure Tu solution. This result can be ascribed to the increased stability of the colloidal particles with CTAB as capping agent. Moreover, this finding indicated that no severe agglomeration of NPs occurred when CTAB was added to the Tu solution. The concentration of the nanoparticles was decreased after adding CTAB, which results in decreasing optical absorption. The agglomerated NPs can be considered as scattering centers in solution, and hence the optical absorption of Ag_2_S increased [30]. A small absorption peak was detected at 355 nm for Ag_2_S prepared with CTAB due to quantum size effects [31]. The absorption of the Ag_2_S NPs decreased sharply above λ = 302 nm for Ag_2_S prepared in pure Tu solution, while it decreased slowly for Ag_2_S prepared in Tu with CTAB, indicating different absorption edges. The optical band gap of the Ag_2_S NPs prepared in pure Tu and Tu with CTAB was calculated by using a Tauc plot.

As shown in Figure 5b, the extrapolation of the linear part of the curve to the photon energy axis produces the optical energy gap. The energy gap of Ag_2_S NPs synthesized in Tu and Tu with CTAB was 1.5 and 2 eV, respectively. The energy gap was increased and a blueshift of about 0.5 eV was observed after CTAB was added to the Tu solution [32]. Furthermore, CTAB prevented particle agglomeration, thereby facilitating the formation of monodisperse Ag_2_S NPs. The value of the obtained energy gap was larger than that of bulk Ag_2_S due to quantum size effects [33]. The particle size (*d*) was calculated from the optical properties using the effective-mass model as shown in Equation 2:

[2]
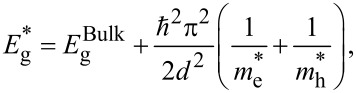


where *E*_g_^*^ is the energy gap of Ag_2_S NPs and *E*_g_^Bulk^ is the energy gap of bulk Ag_2_S, *m*_e_* is the effective mass of an electron and *m*_h_* is the effective mass of a hole. A value of 0.23*m*_o_ for monoclinic Ag_2_S was used for *m*_e_* and *m*_h_* [34]. After substituting the value of energy gap in Equation 2, the particle size of Ag_2_S prepared in pure Tu and Tu with CTAB was 19 and 13 nm, respectively. Increasing the optical energy gap of Ag_2_S NPs can be attributed to the smaller size of the product after adding CTAB.

**Figure 6:**
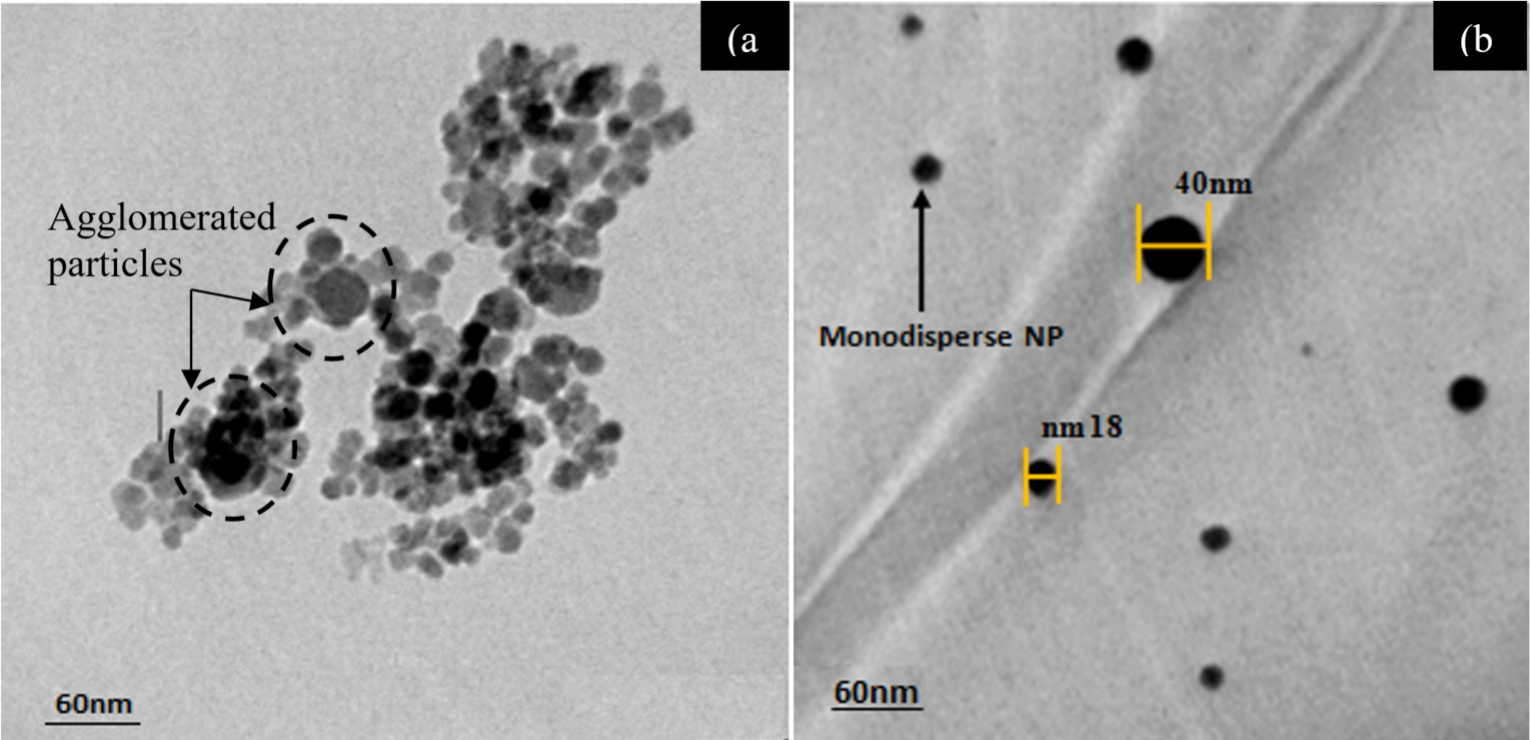
TEM images of Ag_2_S NPs synthesized (a) without CTAB and (b) with CTAB surfactant.

TEM images of the Ag_2_S NPs synthesized with and without CTAB are shown in Figure 6. The TEM image shown in Figure 6a confirms that the Ag_2_S particles prepared in pure Tu had a spherical morphology and different sizes due to the agglomeration effect of the high surface energy of the NPs. The surfaces of spherical particles have high-index crystallographic planes, which increase the surface energy of synthesized NPs [35]. The average particle size was approximately 40 ± 5 nm, and the agglomerated particles tended to form large particles. The Ag_2_S NPs prepared in the mixture of Tu and CTAB were monodisperse NPs with purely spherical shapes, and the average particle size was approximately 30 ± 5 nm. Figure 6b shows monodisperse NPs, which did not agglomerate or aggregate after the CTAB surfactant was added. These observations confirmed the effective capping of CTAB on the Ag_2_S NP surfaces. The reason for the formation of monodisperse Ag_2_S NPs when CTAB was used as a surfactant can be ascribed to the deposition of CTAB on the Ag_2_S NP surface, which resulted in a certain repulsive force to other Ag_2_S NPs. This force may have prevented the agglomeration of the Ag_2_S NPs. CTAB has a positive surface charge [36], and CTAB molecules accumulated on the surfaces of the Ag_2_S NPs and repelled other Ag_2_S NPs due to the mobile electronic charges in Ag_2_S (negative surface charge).

**Figure 7:**
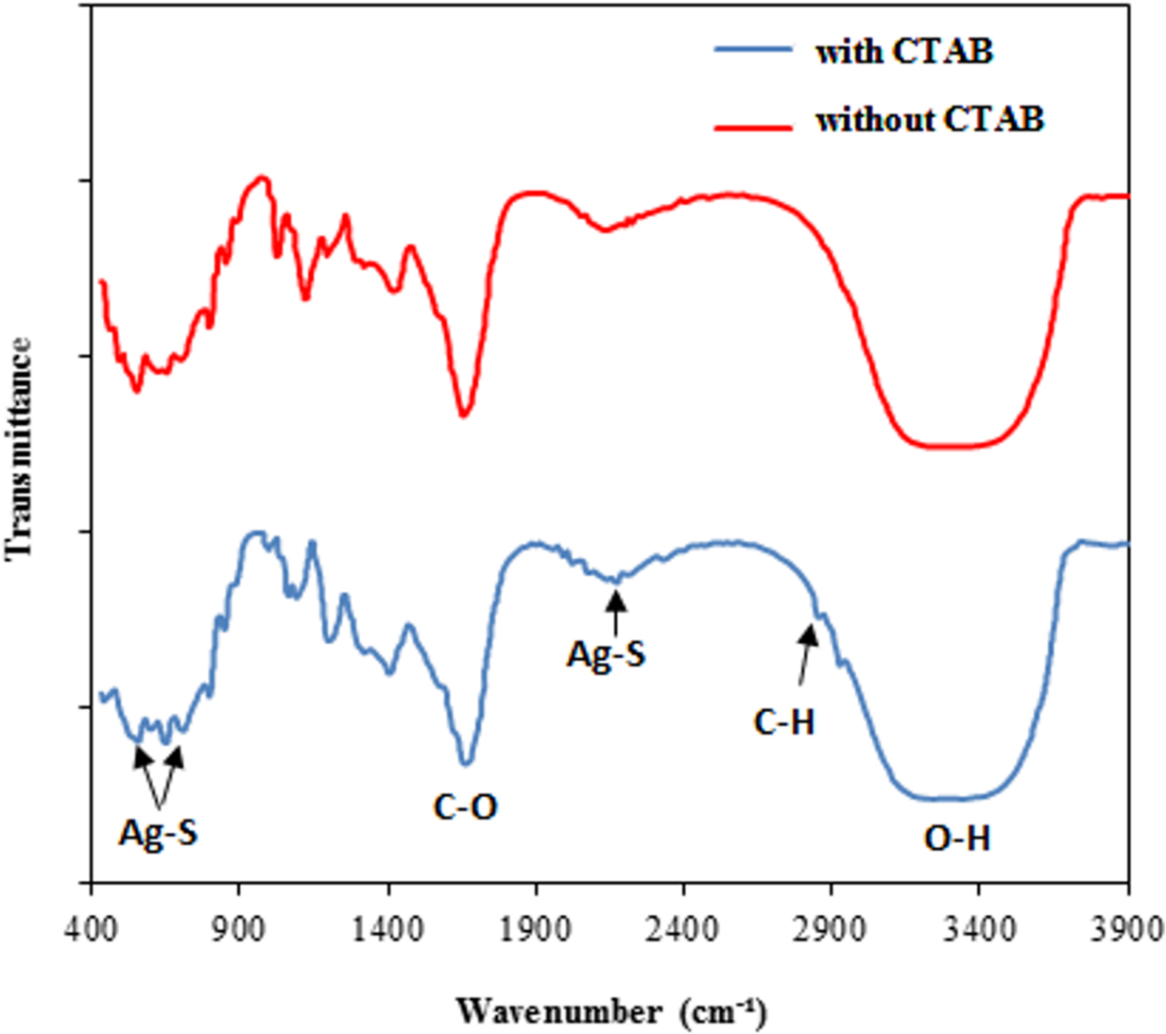
FTIR spectra of Ag2S NPs prepared without and with CTAB.

The FTIR spectra of the Ag_2_S NPs prepared with and without the CTAB surfactant measured in the range of 500–4000 cm^−1^ are shown in Figure 7. The peaks at 541, 640, and 2210 cm^−1^ were indexed to the characteristic vibration of the Ag–S bond. The peak at 1460 cm^−1^ can be indexed to C–C stretching vibration, and the peak at 1650 cm^−1^ belonged to the stretching vibration of the sulfide group. Two peaks appeared at 2842 and 2942 cm^−1^ in the FTIR spectrum of Ag_2_S prepared with CTAB. These peaks were attributed to the methylene (–CH_2_) extension vibration, indicating the adsorption of CTAB on the nanostructure surface. The broad IR peak at 3400 cm^−1^ is indexed to the presence of adsorbed water molecules [37].

**Figure 8:**
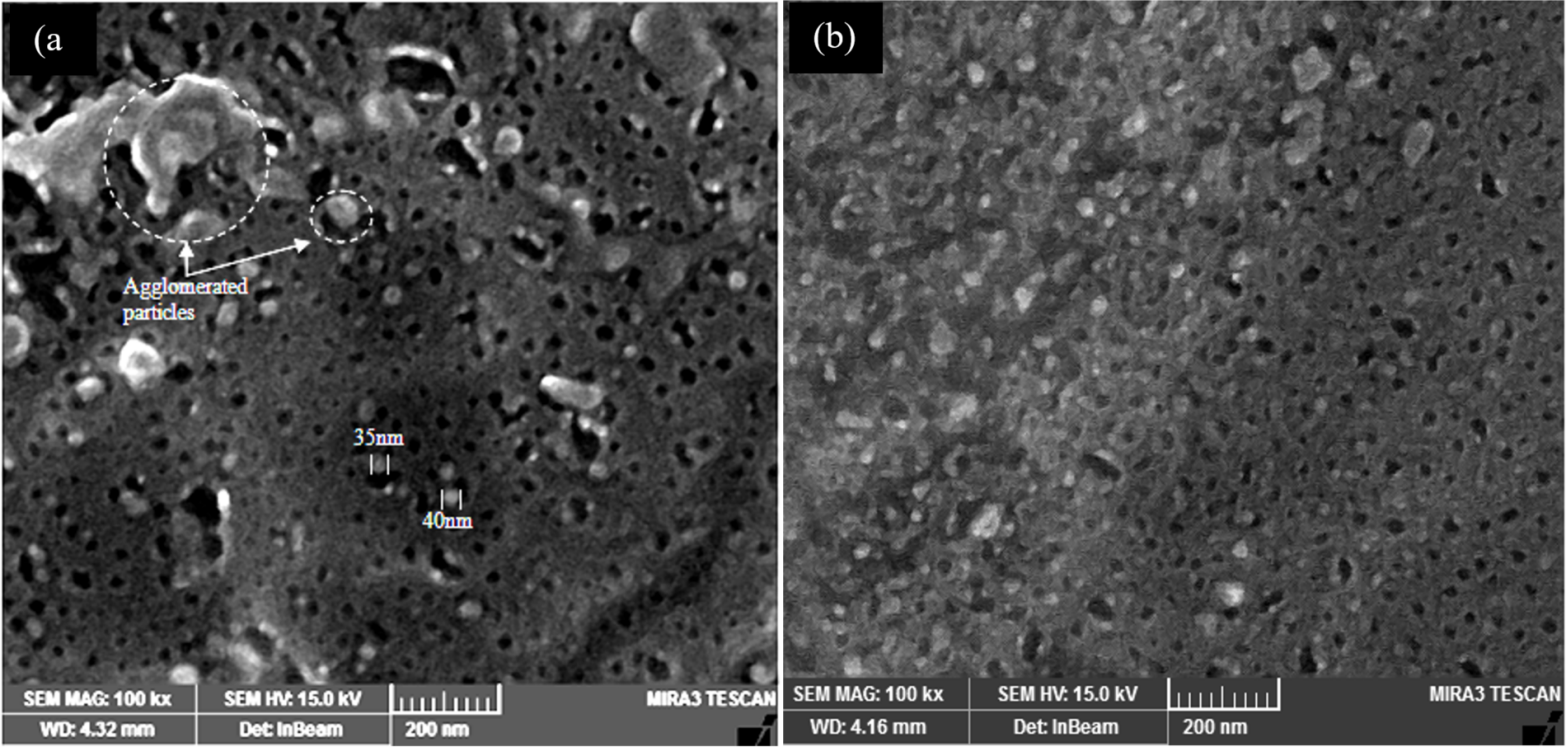
SEM images of Ag_2_S NPs synthesized in pure Tu solution (a) and Tu with CTAB surfactant solution (b).

**Figure 9:**
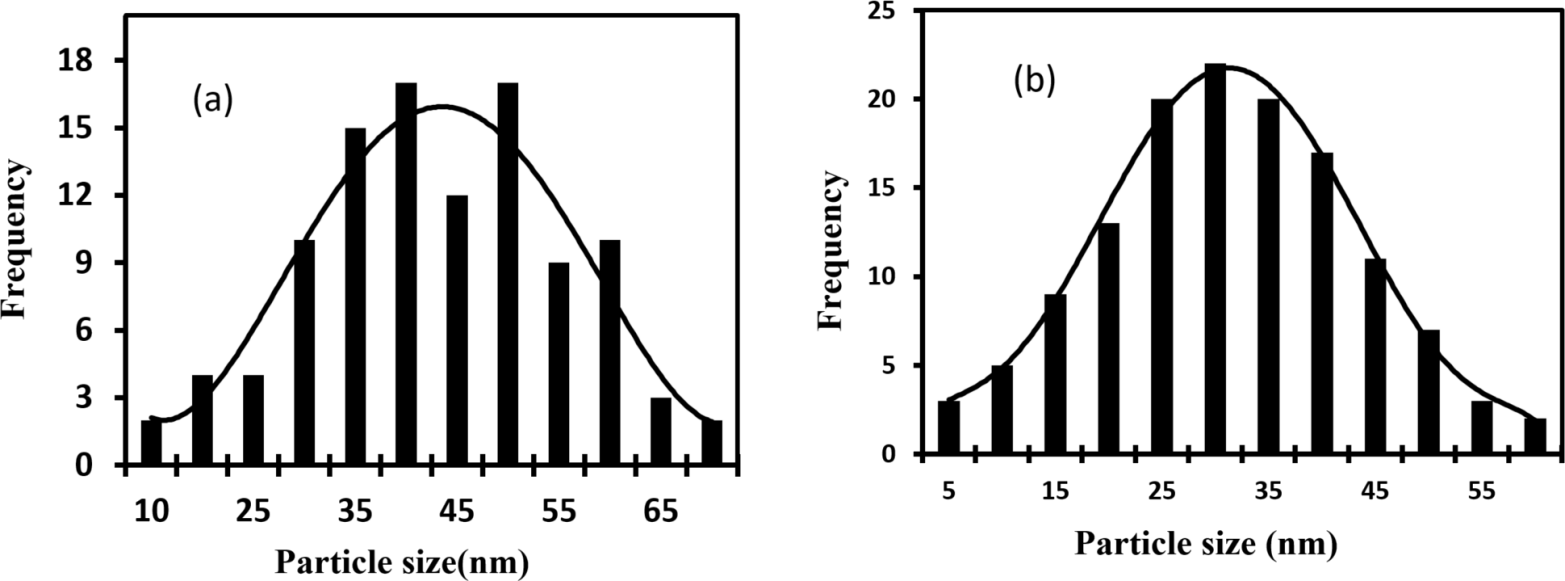
Particle size distribution of Ag_2_S NPs synthesized (a) without and (b) with CTAB.

**Figure 10:**
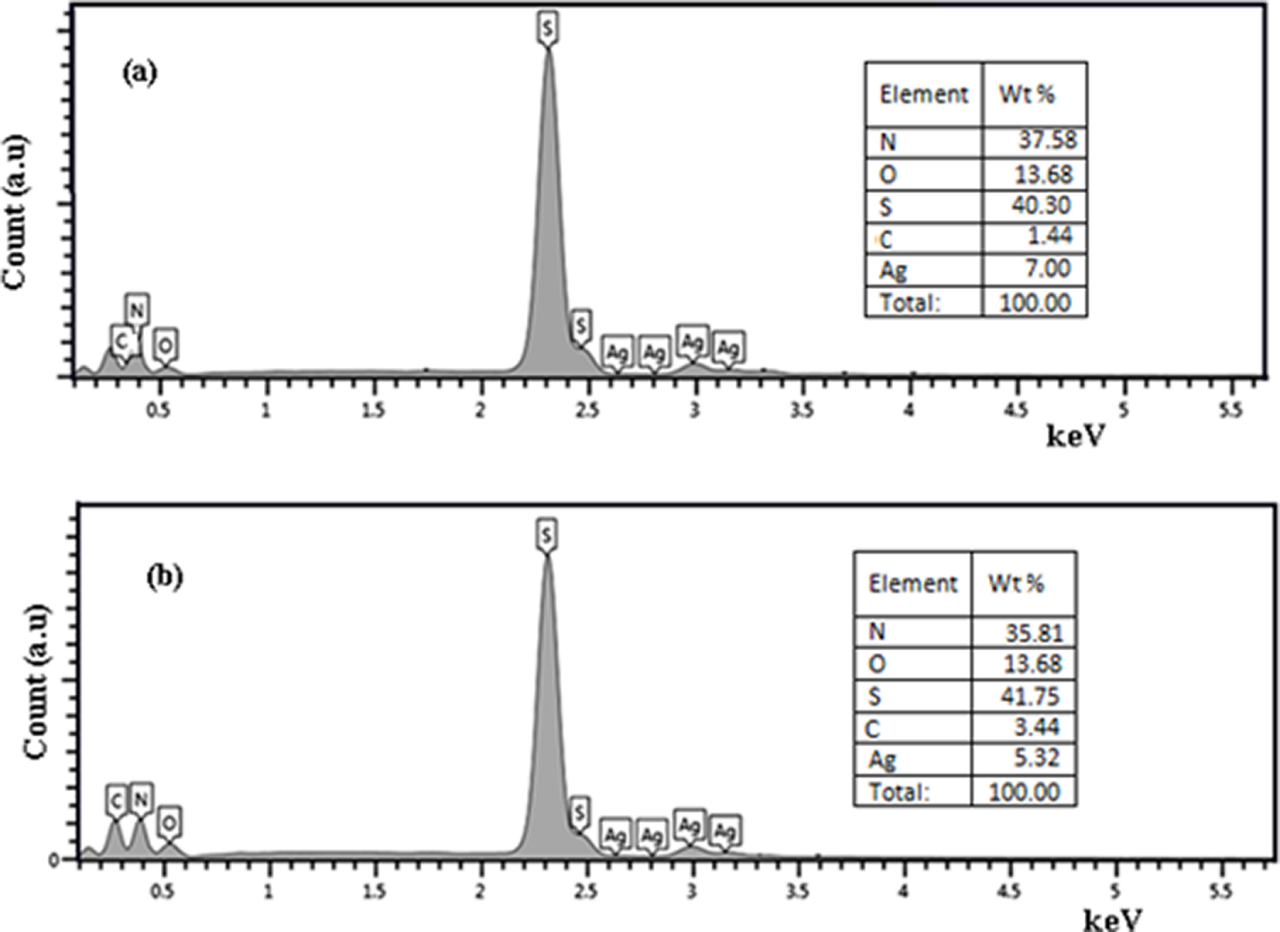
EDX of Ag_2_S NPs synthesized in (a) pure Tu and in (b) Tu with CTAB. The inset shows the measured elements.

Figure 8 shows the SEM images of Ag_2_S NPs synthesized without and with CTAB surfactant. The SEM image of Ag_2_S prepared without CTAB (Figure 8a) shows the formation of agglomerated and aggregated NPs with particle size of 55 nm. The SEM image shown in Figure 8b confirms that when using CTAB surfactant, monodispersed Ag_2_S nanoparticles can be obtained with an average size of 45 nm with few agglomerated NPs. Figure 9 shows the particle size distribution of Ag_2_S synthesized without and with CTAB. The particle size of Ag_2_S NPs ranged from 10 to 70 nm with an average of 55 nm, while the particle distribution of Ag_2_S NPs prepared with CTAB ranged from 5 to 60 nm with an average of 45 nm. The particle size distribution of Ag_2_S prepared with CTAB is nearly Gaussian. The particle size distribution improved after adding CTAB, which plays a major role in preventing particle agglomeration [38]. The energy-dispersive X-ray spectra of the Ag_2_S NPs prepared with and without CTAB are shown in Figure 10.

**Figure 11:**
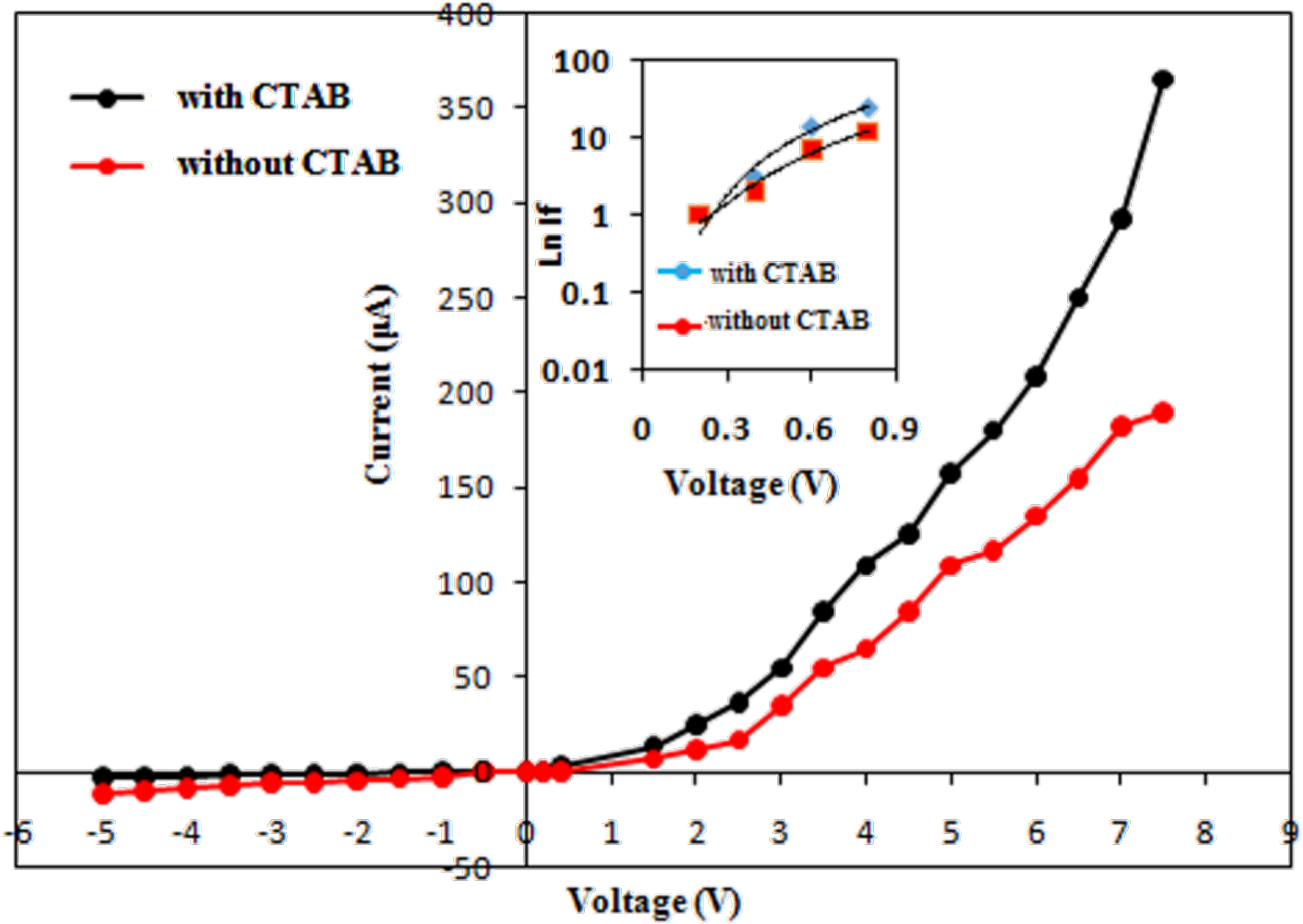
Dark *I*–*V* characteristics of n-Ag_2_S/p-Si heterojunction prepared with and without CTAB. Inset is the semi-logarithmic of forward versus voltage plot.

Figure 10 shows that the [Ag]/[S] weight ratio decreased after adding CTAB. CTAB covered the nanoparticles and led to a decreasing ratio between Ag and S. The origin of nitrogen are thiourea traces attached to the Ag_2_S nanoparticles, while the origin of carbon is CTAB. Hall measurement revealed that the Ag_2_S had a negative Hall coefficient, indicating that it is an n-type semiconductor. This finding agrees well with [39]. Figure 11 shows the dark *I*–*V* characteristics of an n-Ag_2_S/p-Si heterojunction prepared without and with CTAB under forward and reverse current flow. The forward current flow increased exponentially with bias voltage for the Ag_2_S/Si heterojunction synthesized in the presence of the CTAB surfactant, indicating the dominance of diffusion current. In the case of the heterojunction prepared without CTAB, the forward current increased linearly with voltage and tended to saturate at a voltage of 8 V due to the effect of series resistance. The forward current increased after adding CTAB due to the reduced electrical resistance of Ag_2_S. The reverse current of the heterojunction prepared in pure Tu slightly increased with bias voltage, whereas the reverse current of the heterojunction prepared with CTAB did not depend on the bias voltage. These results indicated an enhancement of the junction properties.

**Figure 12:**
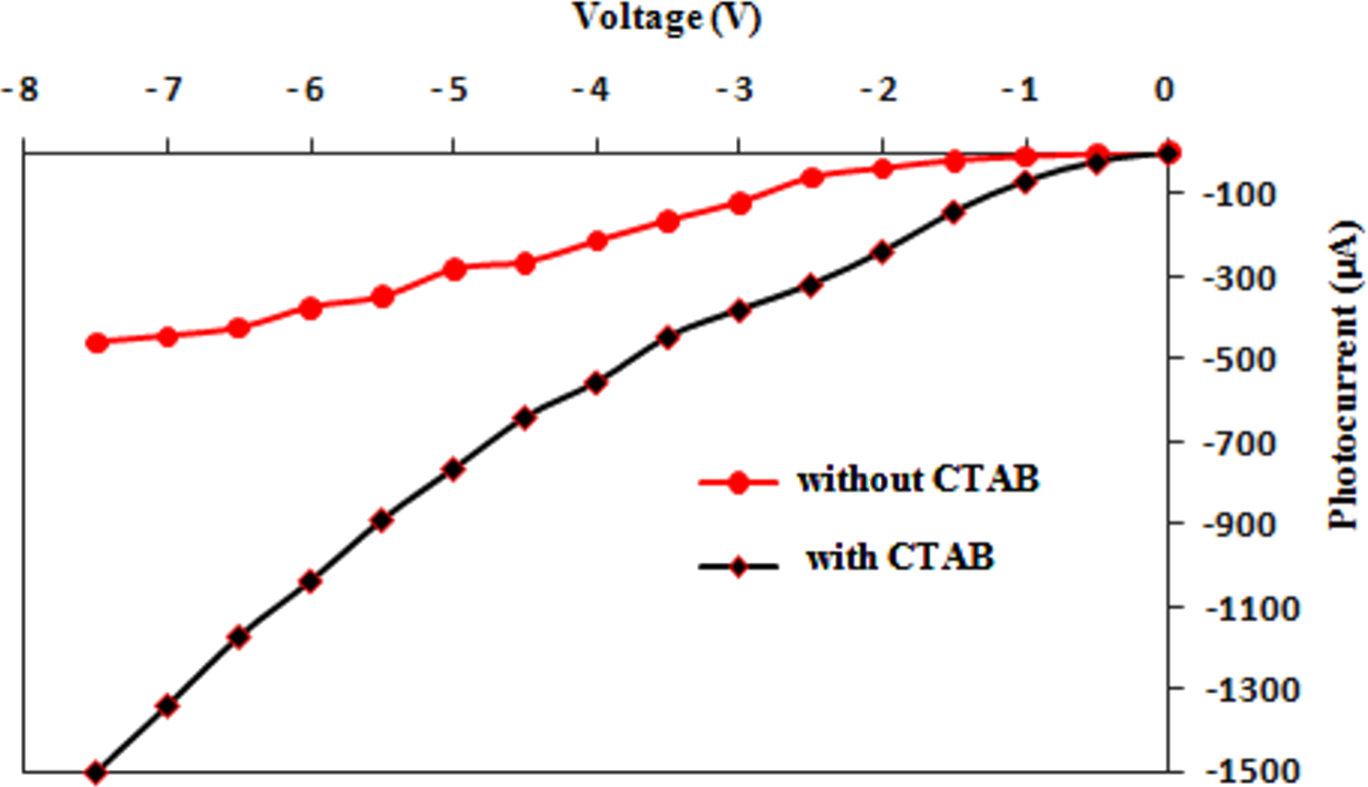
Effect of CTAB on the *I*–*V* characteristics under illumination of the heterojunctions.

The ideality factor (*n*) of Ag_2_S/Si was estimated from the semi-logarithmic plot of the forward current as a function of the voltage (inset of Figure 11) using Equation 3:

[3]
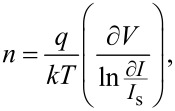


where *I*_s_ is the saturation current of the heterojunction. The value of *n* for the heterojunctions prepared in Tu and Tu with CTAB was 4 and 2.7, respectively. The value reduction of *n* after adding CTAB indicated a remarkable improvement in junction characteristics. The value of turn-on voltage of the heterojunctions was estimated and found to be 2.2 and 1.8 V for heterojunctions prepared in pure Tu and with CTAB surfactant, respectively. Decreasing the turn-on voltage after adding the CTAB can be ascribed to a decrease in the electrical resistivity of Ag_2_S. Figure 12 illustrates the *I*–*V* characteristics under illumination of the heterojunctions at reverse bias. The photocurrent of the heterojunction increased from 460 to 1500 μA at 7.5 V after CTAB was added to the Tu solution.

This result can be ascribed to the positive role of CTAB in increasing the depletion layer width and decreasing the e–h recombination process. The calculated on/off ratio at 5 V of the heterojunctions synthesized without and with CTAB was 42 and 517, respectively. The responsivity *R*_λ_ of the photodetector represent one of the most important figures of merit of the photodetector and it can be defined as the ratio of photocurrent *I*_ph_ to the incident light power *P*_s_ as shown in the following equation:

[4]
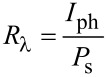


**Figure 13:**
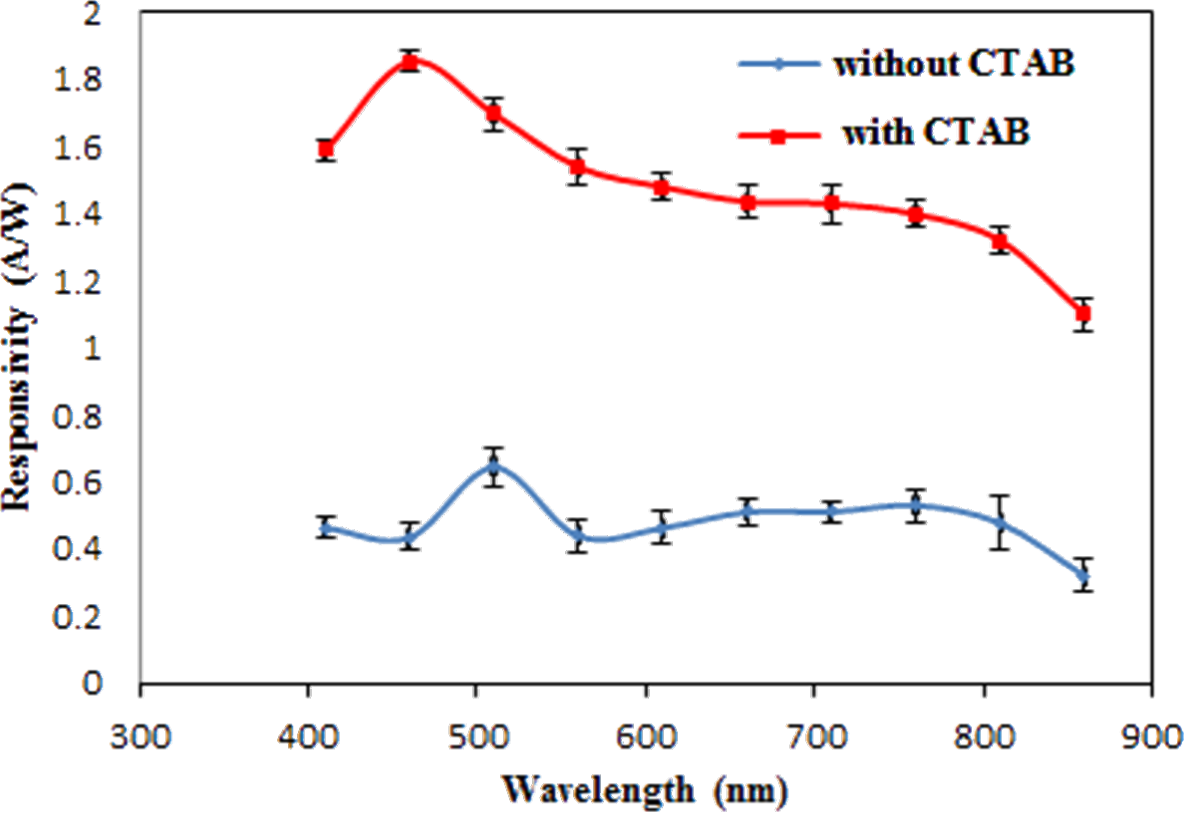
Spectral responsivity Ag_2_S/Si prepared with and without CTAB surfactant.

Figure 13 shows the spectral responsivity (*R*_λ_) of Ag_2_S prepared in the Tu solutions with and without the CTAB surfactant. We observed a response peak at 460 nm, with a responsivity of approximately 1.85 A/W, for the photodetector prepared with CTAB. The responsivity of the photodetector synthesized in pure Tu was 0.64 A/W at 510 nm. The eternal quantum efficiency EQE of the photodetectors prepared without and with CTAB was estimated and found to be 1.5 × 10^2^% and 4.5 × 10^3^% at 510 nm, respectively. This significant increase in the responsivity and quantum efficiency of the photodetector after CTAB addition can be ascribed to the widened depletion layer width and increased minority carrier diffusion length as well as to the large surface area. The blue shift in responsivity of the photodetector after adding of the CTAB can be attributed to the increased energy gap of Ag_2_S NPs. A semi-flat response was observed after 600 nm, and a broad peak was detected at 760 nm.

On the basis of these results, we suggest that adding a surfactant, such as cationic CTAB, to Tu solutions decreases the number of recombination centers and thus reduces the possibility of carrier recombination [39]. The obtained responsivity in the visible region for the photodetector prepared with CTAB was higher than that of silicon-based heterojunction photodetectors [40–44]. The specific detectivity D* of the photodetector was estimated using the following Equation 5:

[5]
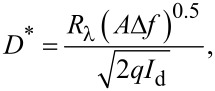


where *A* is the area of the photodetector, Δ*f* is the bandwidth and *I*_d_ is the dark current of the photodetector. The value of detectivity of the photodetector was increased from 0.32 × 10^12^ to 2.3 × 10^12^ Jones at 510 nm and at bias voltage of 1.8 V. Table 1 lists the main figures of merit of Ag_2_S NPs/Si photodetectors prepared without and with CTAB and compared to other photodetectors based on silicon heterojunctions. The fabricated photodetector with CTAB has high figures of merit compared to other photodetectors based silicon heterojunctions.

**Table 1:** Figures of merit of Ag_2_S/Si photodetector and other silicon heterojunction photodetectors.

photodetector type	*R* (A/W)	D* (Jones)	EQE (%)

Ag_2_SNPs/Si without CTAB* (this work)	0.64 at 510 nm	3.2 × 10^11^ at 510 nm	1.5 × 10^2^ at 510 nm
Ag_2_SNPs/Si with CTAB* (this work)	1.85 at 460 nm	2.3 × 10^12^ at 10 nm	4.5 × 10^3^ at 510 nm
Ag_2_SQD/Si [45]	70 μA/W at 1.55 μm	10^11^ at 1.55 μm	–
CdS/Si [46]	0.59 mA/W at 1064 nm	1.3 × 10^12^ at 1064 nm	–
CdTe/Si [47]	0.5 at 950 nm	1.2 × 10^11^ at 950 nm	65 at 950 nm
CdO/Si [48]	0.3 at 600 nm	7 × 10^11^ at 600 nm	62 at 600 nm
InSb/Si [49]	0.132 at 635 nm	1.9 × 10^12^ at 635 nm	–

The main figures of merit of the photodetectors at peak response were investigated after three weeks of storage under ambient conditions (Table 2). There is no significant variation (about 4%) in the values of figures of merit. This indicates that the photodetectors have good environmental stability and they do not need any encapsulation. The photodetector prepared with CTAB exhibits better stability than that prepared in the absence of CTAB. This result is probably due to the high surface activity of the nanoparticles prepared with CTAB.

**Table 2:** Stability of the photodetectors.

time of measurements	*R* (A/W)	D* (Jones)	EQE (%)

immediately			

Ag_2_S/Si (without CTAB)	0.64	3.2 × 10^11^	1.5 × 10^2^
Ag_2_S/Si (with CTAB)	1.8	2.3 × 10^12^	4.5 × 10^3^

after three weeks			

Ag_2_S/Si (without CTAB)	0.62 ± 0.1	3 × 10^11^ ± 0.3	1.42 × 10^2^ ± 0.3
Ag_2_S/Si (with CTAB)	1.72 ± 0.1	2.1 × 10^12^ ± 0.2	4.2 × 10^2^ ± 0.2

**Figure 14:**
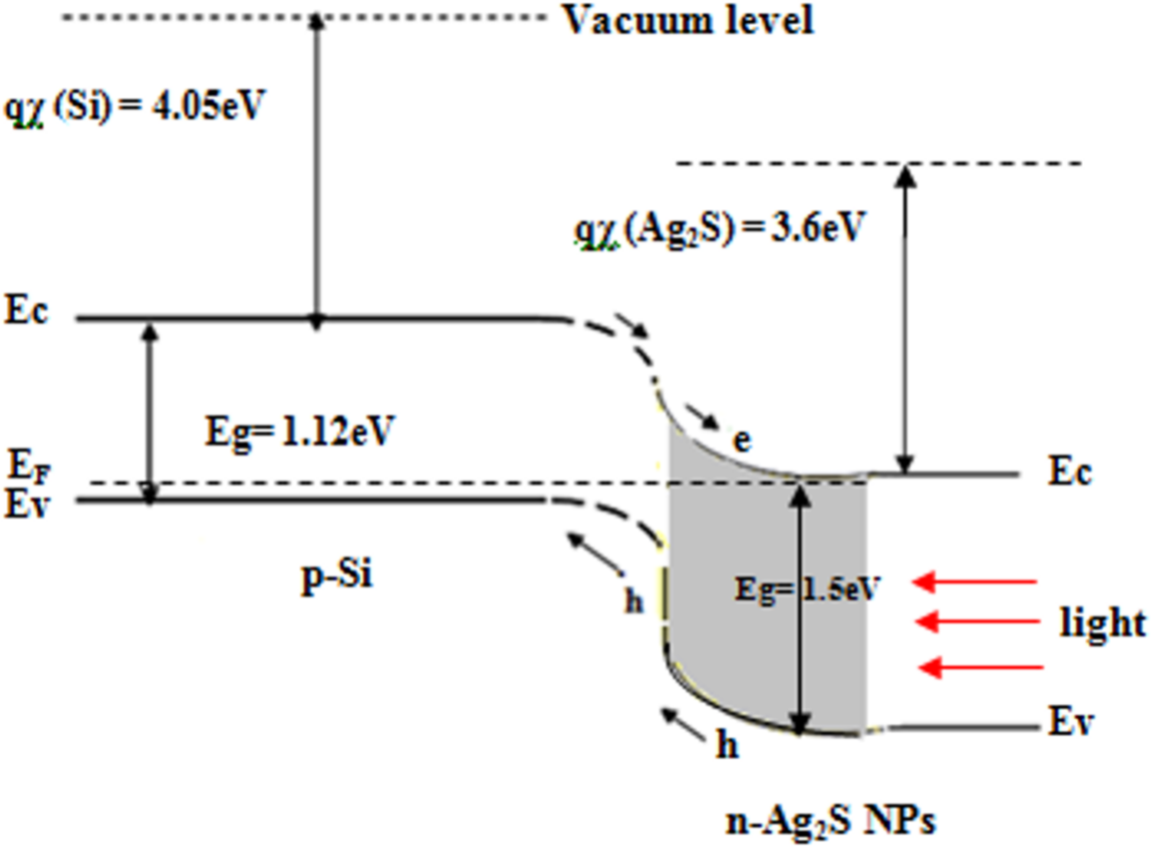
Illuminated energy band diagram of hybrid n-Ag_2_S NPs/p-Si heterojunction.

The energy band diagram under illumination of the n-Ag_2_S NPs/p-Si heterojunction prepared in pure Tu is shown in Figure 14. The electron affinity of Ag_2_S required for the band line-up construction was obtained from reported data [50]. As shown in Figure 12, the photocurrent in the photodetector came from the generated e–h pairs in the depletion region; this process occurred when *h*ν ≥ *E*_g_ (Ag_2_S NPs). As shown in Figure 14, the electrons drifted to Ag_2_S, and the holes diffused toward the p-Si substrate.

## Conclusion

In this work, we successfully prepared monodisperse Ag_2_S NPs by laser ablation of a silver target in a Tu solution with CTAB as a cationic surfactant. The effect of CTAB on the structural, optical and morphological properties of Ag_2_S NPs was studied. XRD results showed a preferred orientation along the (−121) plane after CTAB was introduced. TEM investigation showed that the average particle size was decreased after adding the CTAB surfactant and monodisperse spherical NPs were observed. Energy-dispersive X-ray confirmed the presence of S and Ag elements. The optical energy gap of Ag_2_S increased after adding CTAB surfactant from 1.5 to 2 eV. Raman results indicated the presence of Ag–S bonds and the second-order longitudinal optical phonon 2LO mode; their intensity increased when CTAB was added to Tu. FTIR data revealed the presence of a Ag–S bond located at 510 cm^−1^. The electrical properties of the Ag_2_S/Si heterojunction were significantly enhanced after the addition of CTAB surfactant. The responsivity of the Ag_2_S/Si photodetector at 460 nm was increased by a factor of three after CTAB was added. The energy band diagram of Ag_2_S–Si heterojunction was constructed from optical and electrical results. The photodetectors exhibited good stability, retaining more than 70% of the initial responsivity after storage under ambient conditions for three weeks without any encapsulation. On the basis of these results, the proposed technique is promising and encouraging for the fabrication of inexpensive high-responsivity photodetectors.

## References

[1] Henglein A (1980). J Phys Chem.

[2] Majetich S A, Artman J O, McHenry M E, Nuhfer N T, Staley S W (1993). Phys Rev B.

[3] Storhoff J J, Elghanian R, Mucic R C, Mirkin C A, Letsinger R L (1998). J Am Chem Soc.

[4] Sun Y-P, Riggs J E, Rollins H W, Guduru R (1999). J Phys Chem B.

[5] Sharma R C, Chang Y A (1986). Bull Alloy Phase Diagrams.

[6] Sadovnikov S I, Gusev A I, Rempel A A (2015). Superlattices Microstruct.

[7] Kitova S, Eneva J, Panov A, Haefke H (1994). J Imaging Sci Technol.

[8] Gao F, Lu Q, Komarneni S (2005). Chem Mater.

[9] Yang T, Tang Y, Liu L, Lv X, Wang Q, Ke H, Deng Y, Yang H, Yang X, Liu G (2017). ACS Nano.

[10] Jin R, Cao Y, Mirkin C A, Kelly K L, Schatz G C, Zheng J G (2001). Science.

[11] Ezenwa I, Okreke N A, Egwunyenga N J (2012). Int J Sci Technol.

[12] Aleali H, Sarkhosh L, Karimzadeh R, Mansour N (2011). Phys Status Solidi B.

[13] Sadovnikov S I, Kuznetsova Y V, Rempel A A (2016). Nano-Struct Nano-Objects.

[14] Chen M, Xie Y, Chen H, Qiao Z, Qian Y (2001). J Colloid Interface Sci.

[15] Dong L, Chu Y, Liu Y, Li L (2008). J Colloid Interface Sci.

[16] Zhang C, Zhang S, Yu L, Zhang Z, Zhang P, Wu Z (2012). Mater Lett.

[17] Kang M H, Kim S H, Jang S, Lim J E, Chang H, Kong K-j, Myung S, Park J K (2018). RSC Adv.

[18] Tretyakov I, Shurakov A, Perepelitsa A, Kaurova N, Svyatodukh S, Zilberley T, Ryabchun S, Smirnov M, Ovchinnikov O, Goltsman G (2019). Phys Status Solidi RRL.

[19] Slistan-Grijalva A, Herrera-Urbina R, Rivas-Silva J F, Ávalos-Borja M, Castillón-Barraza F F, Posada-Amarillas A (2005). Phys E (Amsterdam, Neth).

[20] Ismail R A, Khalaf W K, Abdulrazaq O A (2007). Solid-State Electron.

[21] Ismail R A, Ahmed D S, Rawdhan H A (2019). Mater Res Express.

[22] Almeida J M P, Lu C, Mendonça C R, Arnold C B (2015). Opt Mater Express.

[23] Sadovnikov S I, Vovkotrub E G, Rempel A A (2018). Dokl Phys Chem.

[24] Singh Z, Singh I (2019). Sci Rep.

[25] Cifuentes A, Bernal J L, Diez-Masa J C (1997). Anal Chem (Washington, DC, U S).

[26] Cui X, Yuan C, Li S, Hu T, Bao J, Chen S (2017). Micro Nano Lett.

[27] Fu X, Jiang T, Zhao Q, Yin H (2012). J Raman Spectrosc.

[28] Alekperov O, Jahangirli Z, Paucar R (2016). Phys Status Solidi B.

[29] Shakouri-Arani M, Salavati-Niasari M (2014). Spectrochim Acta, Part A.

[30] Mafuné, F, Kohno J-y, Takeda Y, Kondow T, Sawabe H (2000). J Phys Chem B.

[31] Ortega E V, Berk D (2006). Ind Eng Chem Res.

[32] Selvi S S T, Linet J M, Sagadevan S (2018). J Exp Nanosci.

[33] Zhao N, Qi L (2006). Adv Mater (Weinheim, Ger).

[34] Sadovnikov S I, Gusev A I (2017). J Mater Chem A.

[35] Lee S-M, Cho S-N, Cheon J (2003). Adv Mater (Weinheim, Ger).

[36] Arulraj A, Ilayaraja N, Rajeshkumar V, Ramesh M (2019). Sci Rep.

[37] Cava R J, McWhan D B (1980). Phys Rev Lett.

[38] Díaz-Núñez P, González-Izquierdo J, González-Rubio G, Guerrero-Martínez A, Rivera A, Perlado J, Bañares L, Peña-Rodríguez O (2017). Appl Sci.

[39] Abdi S, Dorranian D (2018). Opt Laser Technol.

[40] Zhang J, Liu C, Zhang X, Ke F, Han Y, Peng G, Ma Y, Gao C (2013). Appl Phys Lett.

[41] Chen D, Wei L, Wang D, Chen Y, Tian Y, Yan S, Mei L, Jiao J (2018). J Alloys Compd.

[42] Abdulnabi R K, Mohsin M H, Ismail R A, Mousa A M, Jawad M F (2019). Optik (Munich, Ger).

[43] Ismail R A, Hamoudi W K, Saleh K K (2014). Mater Sci Semicond Process.

[44] Ismail R A, Mousa A M, Shaker S S (2019). Mater Sci Semicond Process.

[45] Tretyakov I, Svyatodukh S, Perepelitsa A, Ryabchun S, Kaurova N, Shurakov A, Smirnov M, Ovchinnikov O, Goltsman G (2020). Nanomaterials.

[46] Dai Y, Wang X, Peng W, Xu C, Wu C, Dong K, Liu R, Wang Z L (2018). Adv Mater (Weinheim, Ger).

[47] Ismail R A, Hassan K I, Abdulrazaq O A, Abode W H (2007). Mater Sci Semicond Process.

[48] Ismail R A, Al-Samarai A-M E, Mohmed S J, Ahmed H H (2013). Solid-State Electron.

[49] Li X, Sun T, Zhou K, Hong X, Tang X, Wei D, Feng W, Shen J, Wei D (2020). Nanotechnology.

[50] Feng Y, Lin S, Wen X, Zhang P, Huang S, Shrestha S, Green M, Conibeer G (2013). Proc SPIE.

